# *In Vivo* Characterization of ARN14140, a Memantine/Galantamine-Based Multi-Target Compound for Alzheimer’s Disease

**DOI:** 10.1038/srep33172

**Published:** 2016-09-09

**Authors:** Angelo M. Reggiani, Elena Simoni, Roberta Caporaso, Johann Meunier, Emeline Keller, Tangui Maurice, Anna Minarini, Michela Rosini, Andrea Cavalli

**Affiliations:** 1 Drug Discovery and Development, Istituto Italiano di Tecnologia, Genova, Italy; 2 Department of Pharmacy and Biotechnology, University of Bologna, Bologna, Italy; 3 Amylgen, Montferrier-sur-Lez, France; 4 INSERM U1198, Montpellier, France; 5 University of Montpellier, Montpellier, France; 6 EPHE, Paris, France

## Abstract

Alzheimer’s disease (AD) is a chronic pathological condition that leads to neurodegeneration, loss of intellectual abilities, including cognition and memory, and ultimately to death. It is widely recognized that AD is a multifactorial disease, where different pathological cascades (mainly amyloid and tau) contribute to neural death and to the clinical outcome related to the disease. The currently available drugs for AD were developed according to the one-target, one-drug paradigm. In recent times, multi-target strategies have begun to play an increasingly central role in the discovery of more efficacious candidates for complex neurological conditions, including AD. In this study, we report on the *in vivo* pharmacological characterization of ARN14140, a new chemical entity, which was obtained through a multi-target structure-activity relationship campaign, and which showed a balanced inhibiting profile against the acetylcholinesterase enzyme and the NMDA receptor. Based on the initial promising biochemical data, ARN14140 is here studied in mice treated with the amyloidogenic fragment 25–35 of the amyloid-β peptide, a consolidated non-transgenic AD model. Sub-chronically treating animals with ARN14140 leads to a prevention of the cognitive impairment and of biomarker levels connected to neurodegeneration, demonstrating its neuroprotective potential as new AD agent.

Alzheimer’s disease (AD) is a chronic neurodegenerative disease characterized by a progressive decline in memory and cognition, leading to loss of body functions and ultimately to death. AD’s etiopathology is largely unknown, although it is widely accepted that the disease is multifactorial, involving parallel and sequential pathological cascades^1^,^2^. In recent years, two major pathways have been reported to be highly involved in disease development: i) the extracellular aggregation of the amyloid beta peptide in protofibrils, fibrils, and plaques, and ii) the intracellular hyperphosphorylation of the tau protein, which is subsequently detached from microtubules with consequent neuronal death^3^,^4^. Oxidative stress and neuroinflammation, with subsequent activation of glial cells, have also been reported to play a role, likely downstream, in AD-associated neurodegeneration^5^,^6^.

Because of its complexity, AD is the source of a major unmet medical need in neurology^7^,^8^. In response to this pluralism of causes and effects, one possible research strategy is to develop a multi-target approach, leading to a single molecule acting in concert on different targets of relevance for the disease. This should allow to gain a superior therapeutic profile, relative to single-target molecules alone, and to overcome the intrinsic conflict of combination therapies, where positive outcomes deriving from combating the disease on multiple fronts coexist with drug-drug interaction concerns^9^. We recently developed a novel class of multi-target compounds, obtained by linking together two commercially available drugs for AD, galantamine and memantine. These drugs modulate the cholinergic and glutamatergic pathways, respectively^10^,^11^. ARN14140 was the endpoint of a mid-sized campaign of dual-target structure-activity relationship (SARs) studies. ARN14140 was the best compromise between pharmacological potency, molecular weight, and calculated logP. When tested *in vitro*, ARN14140 showed a fairly balanced profile against both targets. It was almost equipotent for acetylcholinesterase (AChE) and the NMDA receptor (NMDAR, Fig. 1).

To investigate the effects of ARN14140 in a functional context, we used the intracerebroventricular (i.c.v.) injection of oligomeric amyloid beta peptide 25–35 (Aβ_25-35_) in mice as a rapid, standardized pharmacological model of AD toxicity, which mimics both the cognitive impairment and the associated cellular neurodegeneration^12^,^13^,^14^,^15^,^16^,^17^. Preliminary pharmacokinetic and brain penetration data showed that the compound has a suboptimal profile possibly due to a preferred compound accumulation in the liver (data not shown). To allow brain penetration ARN14140 was given by i.c.v. delivery through osmotic mini pumps providing a continuous flow to interact with the targets. After Aβ_25-35_ intoxication, ARN14140 was delivered for 7 days, releasing an estimated concentration of 12 μM during the treatment. Protection against Aβ_25-35_ neurotoxicity was assessed either behaviorally (spontaneous alternation and passive avoidance) or biochemically (measures of key markers of inflammation and cell integrity).

## Results

Neurodegeneration was induced by i.c.v. injection of Aβ_25-35_. ARN14140 was introduced into the lateral ventricle in mice for 7 days of chronic infusion through an Alzet mini pumps implant. To guarantee full coverage for the entire infusion period, compound doses (2.5 and 7.5 μg/day for 7 days) were selected based on the *in vitro* potency of the molecule on both targets. Since the molecular weight of ARN14140 is 506.73, we estimated a constant concentration of the compound of almost 12 μM. This value was about 6 fold higher than the K_i_ for the NMDA receptor (2.3 μM) and 17 fold higher than the IC_50_ for the AChE enzyme (0.7 μM). Measurements were taken 7–9 days later.

Behavioral readouts included two complementary procedures to assess memory functions. We measured the following biomarkers of neuronal function and integrity: i) hippocampal lipid peroxidation as a cell integrity marker; ii) hippocampal TNFα as a neuroinflammation marker; iii) hippocampal Synaptophysin (Syn) as a synaptic integrity marker. We also looked at Bax and Bcl-2, two proteins involved in apoptotic pathways. Finally, we examined brain tissue integrity to quantify the damage in hippocampal pyramidal cell layers CA1. We assessed the cholinergic loss by immunolabeling of vesicular ACh transporter (VAChT) in cortex, nucleus basalis magnocellularis (Meynert), and hippocampal formation.

### Behavioral tests

**Figure 2:**
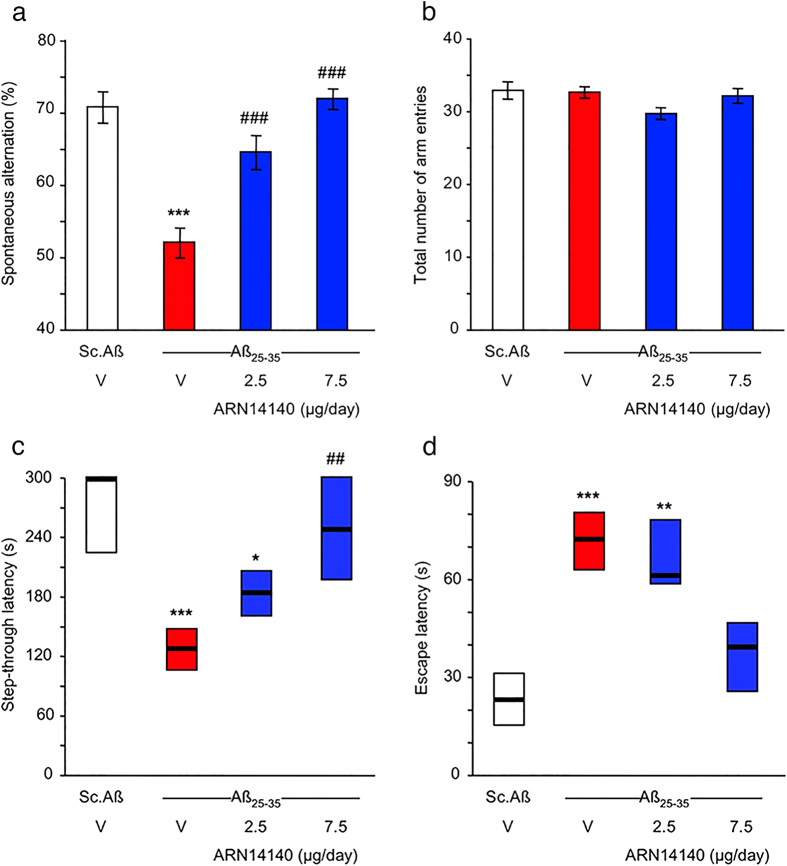
Effects of ARN14140 infusion for 7 days on Aβ_25-35_-induced spontaneous alternation deficits in mice. V, vehicle solution. N = 11–12 per group. ANOVA: F_(3,45)_ = 20.1, p < 0.0001 in (**a**), F_(3,45)_ = 1.22, p > 0.05 in (**b**). ***p < 0.001 vs. the Sc.Aβ/V-treated group; ^###^p < 0.001 vs. the Aβ_25-35_/V-treated group; Dunnett’s test in (**a**). Effects of ARN14140 infusion for 7 days on Aβ_25-35_-induced passive avoidance deficits in mice. (**c**) Step-through latency and (**d**) escape latency measured during the retention session. V, vehicle solution. Data show median and interquartile range (25–75%). N = 11–12 per group. Kruskal-Wallis ANOVA: H = 22.8, p < 0.001 in (**c**), H = 20.3, P < 0.001 in (**d**), *p < 0.05, **p < 0.01, ***p < 0.001 vs. the Sc.Aβ/V-treated group; ^##^p < 0.01 vs. the Aβ_25-35_/V-treated group; Dunn’s test in (**c**,**d**).

Figure 2 reports the effect of ARN14140 on Aβ_25-35_-induced spontaneous alternation deficits in mice during the 7 days. Treatment with Aβ_25-35_ induced significant spontaneous alternation deficits when compared to Sc.Aβ (Fig. 2a). ARN14140 dose-dependently reduced the Aβ_25-35_-induced memory deficits with full effect at a dose of 7.5 μg/day. Conversely, treatments did not affect total arm entries as reported in Fig. 2b.

Figure 2 also reports the results of the passive avoidance test. Figure 2c shows that, with respect to untreated mice or those treated with Sc.Aβ, the Aβ_25-35_ treatment induced highly significant deficits in mice performance both as step-through latency and as escape latency during the retention session (Fig. 2c,d, respectively). Treatment with ARN14140 before training dose-dependently reduced the Aβ_25-35_-induced deficits, with significant prevention at a dose of 7.5 μg/day on both parameters (Fig. 2c,d). The ARN14140 effect was not associated with the typical NMDAR antagonist drawbacks such as increased step-through latency or lower shock sensitivity (data not shown).

In summary, the present findings indicate that chronic infusion of ARN14140 in the lateral ventricles fully protects mice from the development of short-term memory deficits after i.c.v. injection of Aβ_25-35_. Since the Y-maze performance mimics spatial learning and is driven at the hippocampal level, it can be concluded that ARN14140 provides a functional neuroprotective effect for this type of memory. ARN14140 was also effective in the passive avoidance test, which is a fear-motivated test classically used to assess short or long-term memory. Therefore, the data also suggest ARN14140 has an effect on this type of short-term memory. Both memory types are typically affected in AD patients^18^.

### Biochemical analyses

We then analyzed the effect of ARN14140 on specific markers related to neurodegeneration to assess whether the functional neuroprotection seen behaviorally could be associated with a cellular neuroprotection.

**Figure 3:**
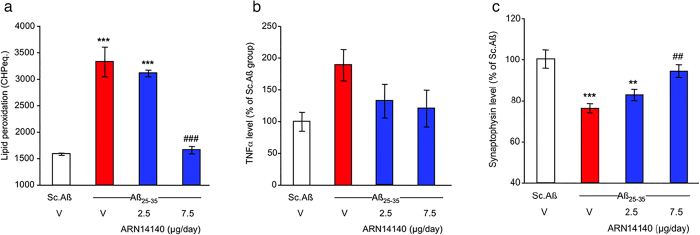
Effects of ARN14140 infusion for 7 days on Aβ_25-35_-induced toxicity. (**a**) Increase in lipid peroxidation in the mouse hippocampus. ANOVA: F_(3,22)_ = 52.2, p < 0.0001, ***p < 0.001 vs. the Sc.Aβ/V-treated group; ^###^p < 0.001 vs. the Aβ_25-35_/V-treated group; Dunnett’s test (**b**) increase in TNFα levels in the mouse hippocampus. ANOVA: F_(3,22)_ = 2.47, p > 0.05. Note that a Dunnett’s test between the Sc.Aβ- and Aβ_25-35_-treated groups led to a significant (p < 0.05) difference (**c**) decrease of synaptophysin level in the mouse hippocampus. ANOVA: F_(3,22)_ = 9.15, p < 0.001, **p < 0.01, ***p < 0.001 vs Sc.Aβ/V, ^##^p < 0.01 vs Aβ_25-35_/V; Dunnett’s test. V, vehicle solution. N = 5–7 per group.

First, we studied lipid peroxidation, a well-known marker of oxidative stress, which is associated with neurodegenerative diseases^19^,^20^. As shown in Fig. 3a, treatment with Aβ_25-35_ induced a highly significant increase in lipid peroxidation in mouse hippocampus compared to Sc.Aβ/V-injected mice. ARN14140 infusion significantly prevented the Aβ_25-35_-induced increase of lipid peroxidation. The effect of ARN14140 was complete at 7.5 μg/day, suggesting that, at this dose, the compound fully reverses the Aβ_25-35_-induced neuronal oxidative damage.

AD has been associated with activation of inflammatory pathways including the hyper secretion of pro-inflammatory, neurotoxic cytokines such as TNFα by reactive microglia and monocytes^21^.

As shown in Fig. 3b, after treatment with Aβ_25-35_, the hippocampal content of TNFα increased compared to ScAβ-injected mice (+89% increase, p < 0.05 using a two-column test, red bar vs. empty bar). As reported in the same figure, continuous i.c.v. infusion of ARN14140 for 7 days tended to prevent the increase in TNFα levels, although the ANOVA failed to reach significance, a similar attenuation was noted with both regimens, 2.5 and 7.5 μg/day. Since TNFα is thought to be part of the initiation mechanism of immune-mediated neurotoxic inflammation^22^, the data suggest that ARN14140 can possibly provide protection against AD-related inflammatory events.

We then investigated the Synaptophysin expression in hippocampus. Syn is a 38 kDa protein, present in virtually all neurons, which participates in synaptic transmission. Reduction of Syn is an index of synaptic function and many reports suggest that Syn levels are lowered by neurodegeneration^23^,^24^.

In our study, treatment with Aβ_25-35_ induced a highly significant decrease of hippocampal Synaptophysin content (−34%) as compared to Sc.Aβ-injected mice (cfr white bar with red bar in Fig. 3c). At a dose of 7.5 μg/day, ARN14140 significantly prevented the Aβ_25-35_-induced decrease in Syn in hippocampus, indicating that, at the top dose, ARN14140 can fully protect synaptic function.

Lastly, we focused on the Bcl-2 protein family. An increase of apoptotic cell death has been reported in the brain tissue of AD patients^25^,^26^. This event has been associated with a dysregulation of the Bcl-2 proteins balance. The Bcl-2 proteins are a family of evolutionarily related proteins mainly involved in regulating programmed cell death (apoptosis). Bax is the most studied member of the family and has pro-apoptotic activity, while Bcl-2 itself is the most investigated family member with anti-apoptotic activity.

**Figure 4:**
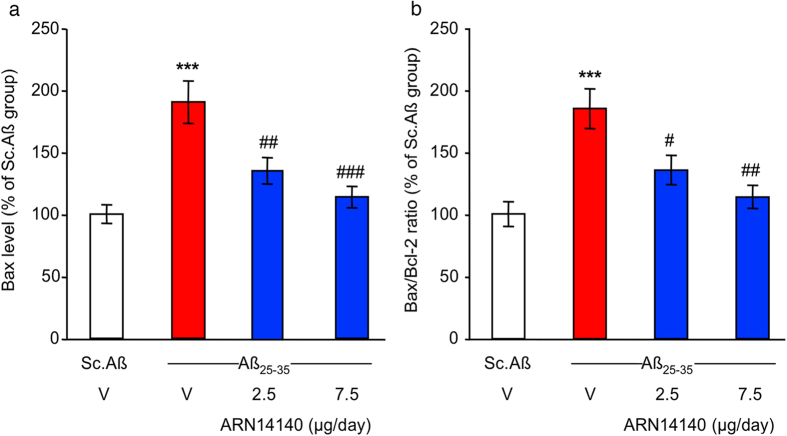
Effect of ARN14140 infusion for 7 days on Aβ_25-35_-induced toxicity. (**a**) Bax level in the mouse hippocampus; (**b**) Bax/Bcl-2 ratio in the mouse hippocampus. V, vehicle solution. N = 5–7 per group in (**a**,**b**), ANOVA: F_(3,22)_ = 12.9, p < 0.0001 in (**a**), F_(3,22)_ = 9.13, p < 0.0001 in (**b**), ***p < 0.001 vs. Sc.Aβ/V, ^#^p < 0.05, ^##^p < 0.01, ^###^p < 0.001 vs. Aβ_25–35_/V; Dunnett’s test. Note that Bcl-2 levels did not differ among groups (F_(3,22)_ = 0.04, p > 0.05).

As reported in Fig. 4a, Aβ_25-35_ administration induced a highly significant increase (+90%) in hippocampal Bax content compared to Sc.Aβ-injected mice. This translates into a highly significant increase (+85%) of Bax/Bcl-2 ratio relative to Sc.Aβ-injected mice (Fig. 4b).

The continuous infusion of ARN14140 for 7 days dose-dependently reduced the increase in Bax content in mouse hippocampus and rebalanced the altered Bax/Bcl-2 ratio (see Fig. 4a,b). In this study, both doses led to a significant effect although the max was observed with the top dose.

In summary, it has been reported that Aβ peptide affect Bax and Bcl-2 expression levels in AD human neurons^27^. Our findings indicate that ARN14140 can rebalance these mechanisms through which can possibly reduce AD-associated apoptosis.

### Morphological alterations

**Figure 5:**
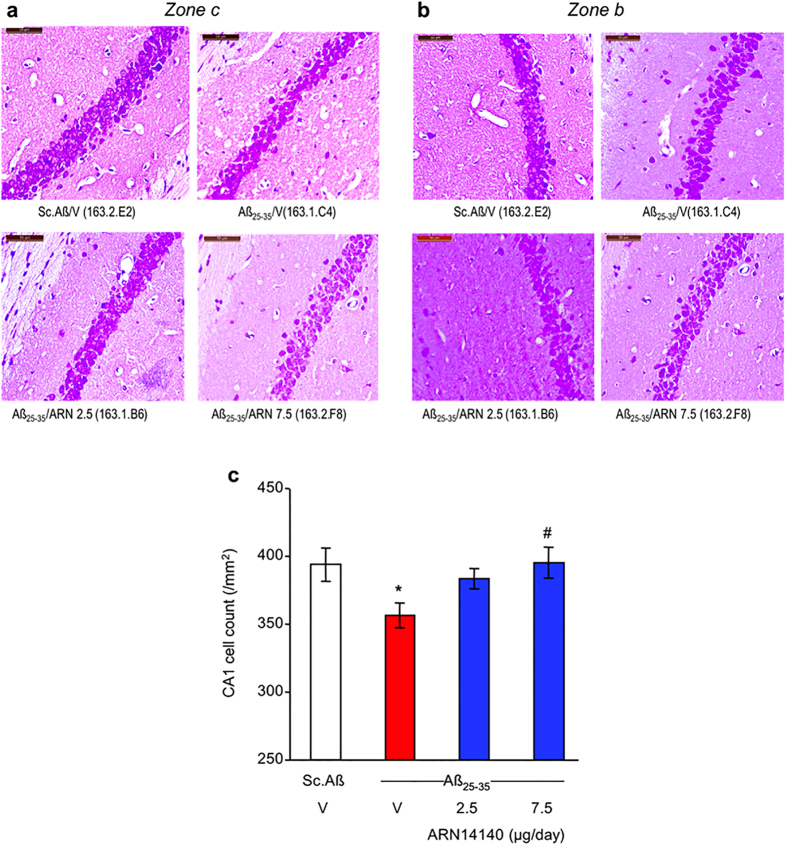
Effects of ARN14140 infusion for 7 days on Aβ_25-35_-induced decrease in viable pyramidal cells in the CA1 layer of the mouse hippocampus. (**a**) Typical CA1 field in zone c; (**b**) typical CA1 field in zone b; (**c**) quantification including zones a–c. V, vehicle solution. N = 10–11 animals per group, with 3 areas counted per animal. ANOVA: F_(3,40)_ = 3.01, p < 0.05, *p < 0.05 vs. Sc.Aβ/V, ^#^p < 0.05 vs. Aβ_25-35_/V; Dunnett’s test. Scale bar = 50 μm in (**a**,**b**).

We studied the neuroprotective potential of ARN14140 at a cellular level by evaluating its effect on Aβ_25-35_ induced CA1 mouse hippocampus cell loss. Cresyl violet was used as a vital stain and a significant decrease in cell count was observed in Aβ_25-35_ treated mice 9 days after i.c.v. injection of the peptides (Fig. 5a,b). The continuous infusion of ARN14140 treatment dose-dependently prevented the effect in viable cells, with a significant difference when compared to the Aβ_25-35_/V-treated group at a dose of 7.5 μg/day (Fig. 5c).

**Figure 6:**
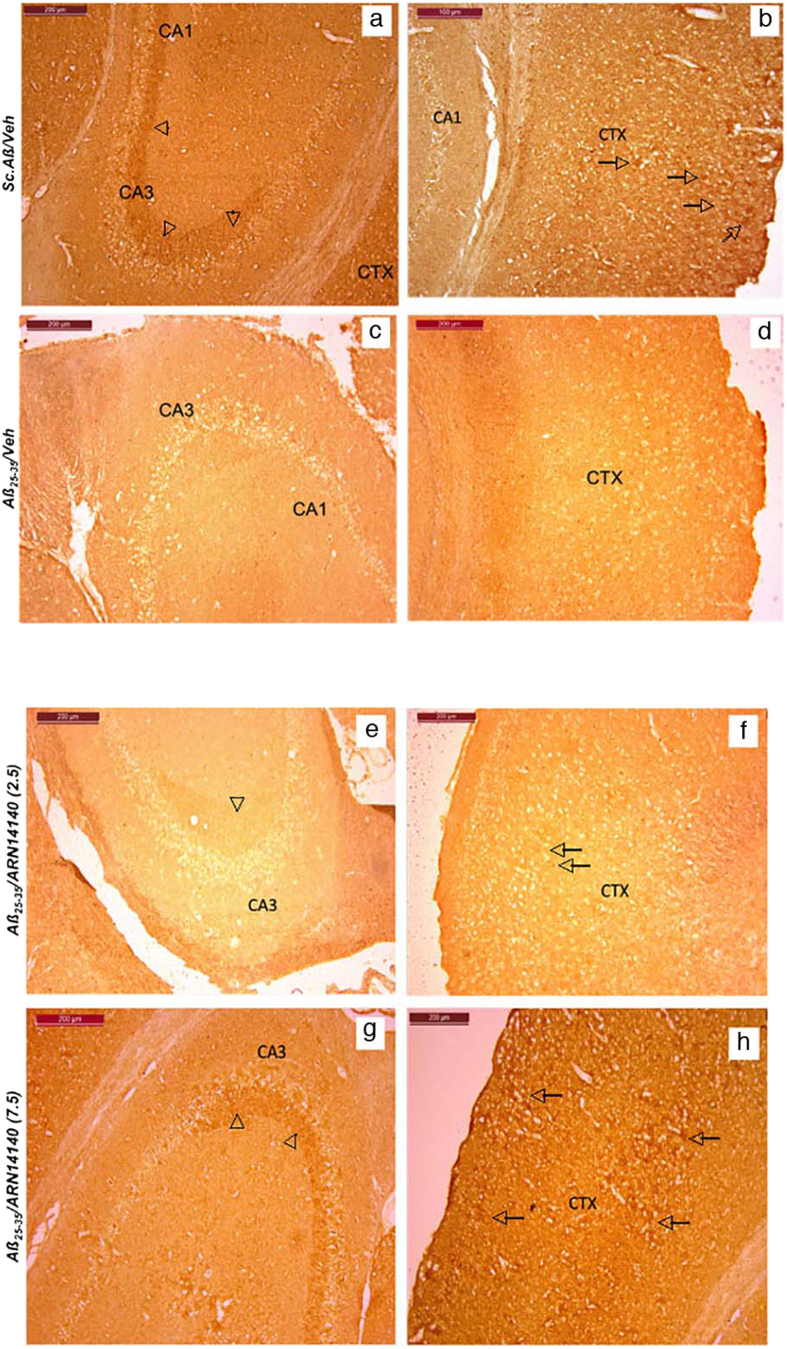
Immunohistochemical staining of VAChT in Aβ_25-35_-treated mouse. Hippocampus (**a**,**c**) and cortex (**b**,**d**) CA3 field in: (**a**,**b**) Sc.Aβ/V-treated mouse and (**c**,**d**) Aβ_25-35_/V-treated mouse. Cholinergic nerve terminals were pointed out on the figures by arrowheads and cholinergic punctae labeling by arrows. Hippocampus (**e**,**g**) and cortex (**f**,**h**) CA3 field in: (**e**,**f**) Aβ_25-35_/ARN14140 (2.5)-treated mouse and (**g**,**h**) Aβ_25-35_/ARN14140 (7.5)-treated mouse. Scale bar = 200 μm.

We then analyzed the acetylcholine marker in different samples. VAChT was stained. VAChT is responsible for loading into neuronal endings ready for secretion. A decrease in VAChT indicates less acetylcholine, most likely due to loss of cholinergic neurons. The qualitative analysis of immunolabeling of VAChT showed large immunoreactive fibers in the hippocampus especially in the CA3 area (see Fig. 6a) and a more punctae labeling in the different cortex layers of Sc.Aβ/V-treated animals (Fig. 6b). In Aβ_25-35_-treated mice, a net decrease in immunolabeling was observed (Fig. 6c,d), which could be attributed to the small loss of neurons measured in the hippocampus.

Poor brain integrity due to the chronic implantation of the pump meant that drug analysis could only be done on qualitative bases (marked glial injury, with high variability among mice). Nonetheless a clear trend of increased density of cholinergic terminals was observed in those mice receiving ARN14140. The effect was detectable at the high dose with an intense labeling of cholinergic terminals in the hippocampus and cortex, as pointed out in Fig. 6g,h.

Our morphological findings confirm at a cellular level that ARN14140 infusion had a beneficial effect on development of neurotoxicity. The effect on CA1 hippocampal cell loss was clear and quantitative, allowing in turn a quantitative analysis of the trend of cholinergic markers.

## Discussion

AD is a complex and multifactorial disease whose causes are still partially unknown. It is widely accepted that AD is associated with a series of concomitant biochemical events that contribute to the pathophysiology of the disease. In the search for more effective therapeutic approaches to AD, an emerging option is to design multi-target molecules. The concept is that multi-target molecules can act at several levels of the AD-related neurodegenerative cascade/s^28^ at the same time. This should provide a profile with greater therapeutic benefits and reduced side effects, compared to single-target compounds.

Pursuing this line of research, we recently designed a novel class of multi-target molecule: dual NMDAR/AChE inhibitors. As starting scaffolds, we used two well-known currently available drugs, galantamine and memantine. We “linked” them into a single compound to form a new class of molecules acting on both targets at the same time. Galantamine was chosen because of its intrinsic dual mechanism of action as an anticholinesterase inhibitor (potentiation of endogenous ACh content) and allosteric modulator of neuronal nicotinic receptors (increase of glutamate levels)^29^,^30^. Memantine was chosen because of its reduced NMDA-related side effects, which may be due to its selective interaction with extrasynaptic NMDAR (selective blockade of extrasynaptic NMDAR does not affect the postsynaptic NMDAR, which is believed to mediate the beneficial glutamate effects in the CNS)^31^.

ARN14140 was the best representative of this new chemical class, which has been the subject of an international PCT patent application (WO2013160728A1).

Based on the initial biochemical characterization, which has already been published^10^, ARN14140 was chosen because it is a fairly balanced molecule. It interacts equally with the AChE enzyme and the NMDA receptor. In this study, the molecule was tested for its ability to prevent the biochemical and behavioral detrimental effects of the i.c.v. injection of Aβ_25-35_. The model is thought to mimic some key phenotypes of AD (mainly cognitive/memory impairment and neurodegeneration), and has already been used to determine neuroprotective effect of galantamine and memantine alone^32^,^33^.

In the present study, ARN14140 was given i.c.v. to minimize all possible issues of brain penetration, since preliminary data showed a suboptimal pharmacokinetic (PK) profile. To maintain a constant inhibition of the AChE and NMDA targets for the required length of time, the compound was given by chronic infusion with osmotic mini-pumps, providing a continuous flow to interact with the targets. For these reasons, ARN14140 is a prototype suitable to explore a concept but insufficient for becoming a medicine. We are now seeking to chemically optimize the PK profile and to explore new delivery strategies, such as transdermal patches, which could minimize the unfavorable plasma profile and maximize ARN14140 therapeutic efficacy.

The Aβ_25-35_-treated animals show a statistically relevant cognitive impairment as well as alteration of the major markers of neurodegeneration and cell death. ARN14140 was able to rescue the behavioral impairment in two different tasks and to balance the levels of biomarkers of neurodegeneration, synaptic plasticity, and apoptosis, which were modified by treatment with Aβ_25-35_. We should recall here that ARN14140 was simultaneously administered to Aβ_25-35_, showing that the molecule could be able to tackle neurodegeneration in the very early stages of the disease. However, based on our data, we could not argue whether ARN14140 is also able to reverse the neuronal damage already present in the brain of AD patients quite in advance of the symptom onset.

When administered to mice for 7 days at a dose of 7.5 μg/day, ARN14140 completely reverted the Aβ_25-35_-induced neurotoxicity in terms of behavioral tasks and biomarkers. We believe this highly positive feature was due to the synergistic potentiation arising from the simultaneous engagement of NMDAR and AChE. To support this hypothesis is the increasing evidence that the glutamatergic and cholinergic neuronal systems influence each other, and that their joint dysfunction is crucial to the effects produced by AD^34^. In particular, based on our recent findings on memantine and galantamine in combination^35^, we may hypothesize that the galantamine moiety could determine AChE inhibition, tackling memory loss and cognitive impairment. In addition, the galantamine function is expected to contrast NMDA-mediated neurotoxicity through the allosteric modulation of nicotinic receptors, thus potentiating memantine’s efficacy in promoting neuronal survival. This interpretation is, however, rather speculative and cannot rule out the involvement of additional, as yet unknown, mechanisms. Our data suggests that ARN14140 could serve as the initial prototype for a pharmacological advancement with respect to the current AD therapies, since it merges complementary activities that could otherwise only be achieved by administering a cocktail of molecules^36^.

Overall, the present study further confirms that multi-target compounds can play an important role in the treatment of complex neurological disorders, and may represent a novel paradigm in anti-AD drug discovery research.

## Methods

### Animals and treatment groups

All experiments were carried out in Amylgen facility (Montpellier, France). Animal procedures were carried out in strict adherence to the European Community Council Directive of September 22, 2010 (2010/63/UE). All experiments and protocols were authorised and approved by the French Ministry of Research, as well as by the Regional Animal Welfare Committee. All efforts were made to minimise the number of animals used.

A total of 72 male Swiss mice (RjOrl:SWISS, Janvier, Saint Berthevin, France) aged 6 weeks old and weighing 30–35 g, were used for Aβ_25-35_ intoxications. Animals were housed in the regulated animal facility of Amylgen (agreement #A 34-169-002 from May 02, 2014), in plastic cages with free access to food and water, except during behavioural experiments. They were kept in a regulated environment under a 12 h light/dark cycle (lights off at 07:00 pm).

### Experimental protocol

Four experimental groups were used, with n = 18 per group: scrambled Aβ_25-35_ (Sc.Aβ) (9 nmol injected intracerebroventricularly (i.c.v.) once) + vehicle solution (infused i.c.v. during 7 days), Aβ_25-35_ (9 nmol injected i.c.v. once) + vehicle solution (infused i.c.v. during 7 days), Aβ_25-35_ (9 nmol injected i.c.v. once) + ARN1410 (infused i.c.v. at 2.5 μg/day during 7 days), and Aβ_25-35_ (9 nmol injected i.c.v. once) + ARN1410 (infused i.c.v. at 7.5 μg/day during 7 days). Animals were kept with n = 8 individuals per cage, the treatment being coded and blind to the experimenter. On day 0, mice were anesthetized using isoflurane 2.5% inhalation. They were injected i.c.v. with Aβ_25-35_ or Sc.Aβ peptide. An Alzet micro-osmotic pump delivering ARN14140 or vehicle solution was placed subcutaneously with the brain cannula into the lateral ventricle. On day 7, the spontaneous alternation performance was tested for n = 12 animals in the Y-maze test, an index of spatial working memory. On days 8 and 9, the contextual long-term memory was assessed using the step-through type passive avoidance procedure for the same animals, with training session on day 8 and retention session on day 9. On day 9, all animals were sacrificed. For half of the animals (n = 6 per group), the hippocampi and frontal cortex were dissected out and frozen in liquid nitrogen before being stored at −80 °C before analyses. One hippocampus was used to measure lipid peroxidation levels in tissue extracts. One cortex was used for ELISA assays. A second set of animals, with n = 6 animal per experimental group, were administered as described and transcardially perfused at day 9 with paraformaldehyde to allow brain slicing and histology/immunohistochemistry. These animals were not tested behaviorally.

### Drugs and administration procedures

The Aβ_25-35_ peptide (SC489) and its control scrambled peptide, containing the 11 amino-acids of Aβ_25-35_ but in a random order, Sc.Aβ (SC492) were purchased from Polypeptides (France). Peptides were solubilized in sterile distilled water at a concentration of 3 mM and the homogeneous oligomeric preparation of Aβ_25-35_ peptides was performed according to Amylgen’s own procedure, leading to a > 98% pure soluble oligomers solution similar as described by Zussy *et al*.^12^,^13^. A quality control analysis for Aβ_25-35_ aggregation was systematically performed by analyzing Sc.Aβ and Aβ_25-35_ solutions using a light scattering assay before each injection of Aβ_25-35_ peptides. The Sc.Aβ solution (3 mM) does not aggregate and must show an OD_320 nm_ < 0.2 while the Aβ_25-35_ solution at the same concentration must show an OD_320 nm_ > 0.8. Each mouse was anesthetized with isoflurane 2.5% and injected i.c.v. with Aβ_25-35_ or Sc.Aβ peptides (9 nmol/mouse) in a final volume of 3 μL per mouse. The injection was performed using the hand-free method described by Haley & McCormick^37^, with the injection coordinates from Bregma: AP −0.4 mm, L 1.0 mm, V −2.5 mm (Paxinos and Franklin, 2012), as previously described^14^,^32^,^38^,^39^,^40^,^41^. ARN14140 was solubilized in PBS/0.5% acetic acid, pH = 6, at a concentration of 13.3 mg/mL and then diluted to the appropriate concentrations. It was infused using Alzet micro-osmotic pumps 1007D delivering 0.5 μL/h over 7 days (total volume 100 μL, solution concentrations of 0.21 g/L and 0.63 g/L). Each pump was connected to a brain infusion kit III (Charles River, L’Arbresles, France). Behavioral studies were carried out blind to the experimenter.

### Spontaneous alternation performances

The spatial working memory was examined by measuring the spontaneous alternation behaviour of the mice in the Y-maze^14^,^32^,^38^,^39^,^40^,^41^. The Y-maze is made of grey polyvinylchloride. Each arm is 40 cm long, 13 cm high, 3 cm wide at the bottom, 10 cm wide at the top, and converging at an equal angle. Seven days after Aβ_25-35_ or Sc.Aβ peptide injection, each mouse was placed at the end of one arm and allowed to move freely through the maze during an 8 min session. The series of arm entries, including possible returns into the same arm, was recorded visually. An alternation was defined as entries into all three arms on consecutive occasions. The number of maximum alternations was the total number of arm entries minus two and the percentage of alternation was calculated as actual alternations/maximum alternations × 100. Measured parameters included the percentage of alternation (memory index) and total number of arm entries (exploration index).

### Passive avoidance test

The contextual long-term memory of the animals was assessed using a two-session step-through passive avoidance procedure^32^,^40^,^41^.

The apparatus consisted of two compartments (15 × 20 × 15 cm high), one illuminated with white polyvinylchloride walls and the other darkened with black polyvinylchloride walls and a grid floor for electrical shocks. A guillotine door separated each compartment. A 60 W lamp positioned 40 cm above the apparatus lights up the white compartment during the experiment. Scrambled foot shocks could be delivered to the grid floor using a shock generator scrambler (Lafayette Instruments, Lafayette, USA). Training: 8 days after Aβ_25-35_ or Sc.Aβ peptide injection, each mouse was placed into the white compartment. After 5 s, the door was raised. When the mouse entered the darkened compartment and placed all its paws on the grid floor, the door was closed and the foot shock (0.3 mA) delivered for 3 s. The latency spent to enter the dark compartment and the number of vocalisations was recorded. None of the treatment affected the step-through latency (H = 0.31, p > 0.05) or vocalisations (H = 2.83, p > 0.05) during training. Retention: 24 h after training (9 days after Aβ_25-35_ or Sc.Aβ peptide injection), each mouse was placed again into the white compartment. After 5 s, the door was raised and the step-through latency (latency to enter the dark compartment) was recorded up to 300 s.

### Lipid peroxidation

The quantification of lipid peroxidation in tissue extracts is based on Fe(III)xylenol orange complex formation according to Hermes-Lima *et al*.^42^. Six mice for each group were sacrificed by decapitation, without anesthesia nor sedation, 9 days after Aβ_25-35_ or Sc.Aβ peptide injection. The brains were rapidly removed, weighed and kept in liquid nitrogen until assayed. After thawing, one hippocampus per mouse was homogenized in cold methanol (1/10 w/v), centrifuged at 1,000 *g* for 5 min and the supernatant placed in an Eppendorf tube. The reaction volume of each homogenate was added to FeSO_4_ 1 mM, H_2_SO_4_ 0.25 M, xylenol orange 1 mM and incubated for 30 min at room temperature. After reading the absorbance at 580 nm (A_580_1), 10 μL of cumene hydroperoxide (CHP) 1 mM was added to the sample and incubated for 30 min at room temperature, to determine the maximal oxidation level. The absorbance was measured at 580 nm (A_580_2).

The level of lipid peroxidation was determined as CHP equivalents according to: CHPE = A_580_1/A_580_2 × [CHP (nmol)] and expressed as CHP equivalents per mg of tissue and as a percentage of the control group data (V-treated Sc.Aβ-administered mice).

### Biochemical analyses

Nine days after Aβ_25-35_ or Sc.Aβ injection, 6 animals were killed by decapitation, and hippocampi from 6 animals were dissected out, weighted and immediately frozen in liquid nitrogen. 6 hippocampi were used per group for ELISA assays.

For ELISA analysis, 6 hippocampi were homogenized in 50 mM Tris-150 mM NaCl buffer, pH 7.5 in eppendorf tubes, and sonicated for 2 × 10 s at 4 °C (2 × 1,0 kJ, BANDELIN Sonopuls HD3200). After centrifugation (16100 g for 15 min, 4 °C), the supernatants were assayed immediately by ELISA assay for tumor necrosis factor alpha (TNFα) (EMTNFA01, ThermoScientific), synaptophysin (Syn) (E90425M, USCN), Bax (E91343M, USCN), and Bcl-2 (E90778M, USCN) according to the manufacturer instructions. For each assay, absorbance was read at 450 nm and sample concentration was calculated using the standard curve. Results are expressed in pg of marker per mg of tissue.

Protein concentration was determined in brain homogenates with the BCA protein assay kit (Pierce Perbio Science, France).

### Histology/immunohistochemistry

Nine days after Aβ_25-35_ or Sc.Aβ peptide injection, 6 mice from each group were anesthetised by 200 μL intraperitoneal injection of a premix of ketamine (80 mg/kg) and xylazine (10 mg/kg), and quickly perfused transcardially with 100 mL of saline solution (NaCl 0.9%) followed by 100 mL of paraformaldehyde 4%. Brains were removed and kept for 48 h in the fixative solution at 4 °C, then cut into 10 μm thickness coronal sections using a cryostat. Sections were stained with 0.2% cresyl violet reagent (Sigma-Aldrich), dehydrated with graded ethanol, treated with toluene, and mounted with Mountex medium (BDH Laboratory Supplies, Poole, Dorset, UK).

Examination of the CA1 area was performed using digitalized slices using a Nanozoomer virtual microscopy system (Hamamatsu, Massy, France). CA1 measurement and pyramidal cell counts were processed using a 40 × objective using ImageJ software (NIH). Data were expressed as mean number of viable cells per mm, from at least four slices × 3 CA1 fields × 2 hemispheres for each group, according to the previously reported method^12^.

For immunohistochemical labeling of the vesicular acetylcholine transporter (VAChT), sections were incubated overnight at 4 °C with Rabbit monoclonal anti-VAChT (SOSV00002G, Thermofisher). Then, sections were incubated with Rabbit biotinylated secondary antibody (B8895, Sigma-Aldrich) and incubated for 1 h in avidin-biotin complex (ABC Vector laboratory). Signal was detected with the diaminobenzidine kit (Vector, Laboratories). Examination of the stained area was performed using digitalized slices using Optic microscope (Leica).

### Statistical analyses

All values, except passive avoidance latencies, were expressed as mean ± S.E.M. Statistic analyses were performed on the different conditions using one-way ANOVA (*F* value), followed by the Dunnett’s post-hoc multiple comparison test. Passive avoidance latencies do not follow a Gaussian distribution, since upper cut-off times are set. They were therefore analyzed using a Kruskal-Wallis non-parametric ANOVA (*H* value), followed by a Dunn’s multiple comparison test. *p* < 0.05 was considered as statistically significant.

## Additional Information

**How to cite this article**: Reggiani, A. M. *et al*. *In Vivo* Characterization of ARN14140, a Memantine/Galantamine-Based Multi-Target Compound for Alzheimer’s Disease. *Sci. Rep.* **6**, 33172; doi: 10.1038/srep33172 (2016).

## Figures and Tables

**Figure 1:**
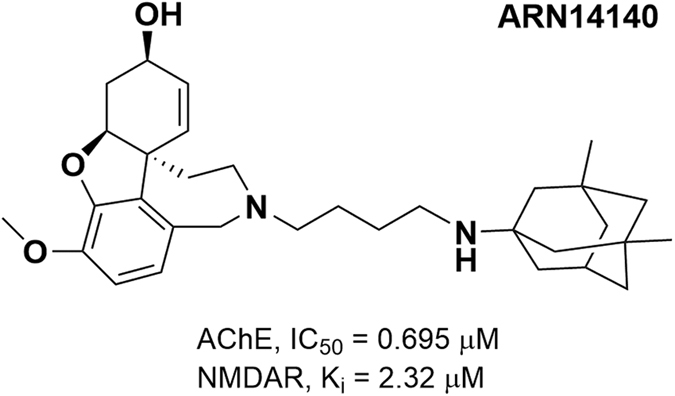
2D structure of ARN14140 (MW = 506.73) along with biological activities values against AChE and NMDAR as reported in Simoni **et al.^10^.
